# The dynamics and functional mechanisms of H2B mono-ubiquitination

**DOI:** 10.1007/s44297-023-00022-9

**Published:** 2024-01-25

**Authors:** Yiqing Li, Tianling Ma, Jinhua Jiang, Matthias Hahn, Yanni Yin

**Affiliations:** 1 State Key Laboratory of Rice Biology, Key Laboratory of Molecular Biology of Crop Pathogens and Insects, Institute of Biotechnology, Zhejiang University, 866 Yuhangtang Road, Hangzhou, 310058 China; 2 Department of Plant Pathology, Zhejiang Agriculture and Forest University, Hangzhou, 311300 China; 3 State Key Laboratory for Managing Biotic and Chemical Threats to the Quality and Safety of Agro-products, Institute of Agro-product Safety and Nutrition, Zhejiang Academy of Agricultural Sciences, Hangzhou, 310021 Zhejiang China; 4 Department of Biology, RPTU University of Kaiserslautern, PO Box 3049, 67653 Kaiserslautern, Germany

**Keywords:** H2B ub1, deub1, Balance maintenance, Transcription, Trans-histone crosstalk, Plant-pathogen interactions

## Abstract

H2B mono-ubiquitination (ub1) is an important histone modification attaching a ubiquitin moiety to the small histone H2B and changing the biochemical features of the chromatin. The dynamic equilibrium between H2B ub1 and deubiquitination (deub1) has been shown to affect nucleosome stability, nucleosome reassembly and higher chromatin structure. The above changes mediated by H2B ub1 regulate transcription activation and elongation, and play key roles in multiple molecular and biological processes including growth, development, pathogenesis and aging. In this review, we summarize our current knowledge in regulation of H2B ub1/deub1 equilibrium, and how this modification affects chromatin dynamics and gene expressions. We also discuss the roles of H2B ub1/deub1 cycle in plant-pathogen interactions, and point out the questions that remain to be resolved in future studies.

## Introduction

Eukaryotes pack their large genomes into chromatin but need to keep important DNA regions accessible for coordinated gene expression. The nucleosome core particle (NCP) is a basic unit of the chromatin polymer, consisting of an octameric complex of core histones (H2A, H2B, H3, and H4) that is encircled by 145–147 bp of DNA [1]. Histones are subject to a variety of post-translational modifications (PTMs) including acetylation, methylation, ubiquitination and phosphorylation that alter the transcriptional properties of chromatin. In contrast to other histone modifications which are concentrated in the unstructured N-terminal tails, H2B mono-ubiquitination (ub1) is embedded within the C-terminal α helix [2, 3], and this hardly accessible modification site may account for the generally low levels of H2B ub1 in chromatin [2–4]. Similar to other histone PTMs, H2B ub1 is reversible and depends on the balanced activity of its writers and multiple editors [5, 6].

Since ubiquitin has roughly half the size/mass of a histone protein, H2B ub1 is expected to exert a major effect on chromatin structure and accessibility [7]. Indeed, numerous biochemical studies have shown that H2B ub1/deub1 function in the modulation of chromatin state [8, 9], nucleosome stability regulation [10–12], trans-histone regulation of histone H3 lysine 4 and lysine 79 methylation [2, 12–16]. Thus this modification is tightly associated with a number of cellular functions including regulation of transcription activation and elongation, by changing the chromatin state between transcriptionally active euchromatin or inactive heterochromatin [7, 9, 17–19]. In this review, we mainly focus on discussing the dynamic modulation of H2B ub1/deub1 states and the regulatory mechanisms that are associated with this modification, including its effects on chromatin dynamics, nucleosome stability, trans-histone crosstalk, transcriptional initiation and elongation and heterochromatin silencing. We will also address the roles of H2B ub1/deub1 in the interactions of plants and their fungal pathogens.

## Dynamic equilibrium between H2B mono-ubiquitination and deubiquitination

### H2B mono-ubiquitination

‘Writers’ are defined as catalytic enzymes that perform modifications on histones [5]. In *Saccharomyces cerevisiae*, the ubiquitin-conjugating enzyme Rad6 and the E3-ligase Bre1 act as H2B ub1 writers by covalently attaching ubiquitin onto K123 [1] (Table 1). Indeed, mutation of the H2B ubiquitination site (H2BK123R), or deletion of either Rad6 or Bre1 led to a complete disappearance of H2B ub1 [20, 21]. Besides the writers, H2B ub1 is also elaborately regulated by other catalytic factors with complex mechanisms. Lge1, a cofactor of Bre1 was reported to be required for full level H2B ub1 [18], and its stability controlled by Bre1 is crucial for H2B ub1 regulation [22] (Table 2). Similarly, the Bur1/Bur2 complex mediating phosphorylation of serine 120 of Rad6 has been reported to be critical for full H2B ub1 [23] (Table 2). Besides, the Rtf1 subunit of the polymerase associated factor 1 (Paf1) complex was found to regulate Bre1 stability and to be necessary for fine control of H2B ub1 levels [24] (Table 2). Moreover, the histone modification domain (HMD) of Rtf1 was shown to directly interact with Rad6 and to increase H2B ub1 [25] (Table 2). Similarly, in *Schizosaccharomyces pombe*, the Rad6 homologue ubiquitin-conjugating enzyme Rhp6 forms a multi-subunit protein complex (HULC) with two RING finger proteins Rfp1 and Rfp2, both sharing homology with budding yeast Bre1 (Table 1), and a novel serine-rich protein Shf1 [8]. The HULC complex is required for H2B ub1 at lysine 119 (homologous to *S. cerevisiae* lysine 123) both in vivo and in vitro [8] (Table 2). Additionally, the activity of ubiquitin ligase Brl1 was reported to be regulated by acetylation driven by Mst2, a member of the MYST family of histone acetyltransferases (Table 2). Mst2 acetylates Lysine 242 of Brl1, and there is an overall reduction of H2B ub1 level in Brl1-K242R cells (Brl1 cannot be acetylated) compared to the wild type [26]. In mammalian cells, H2B ub1 is performed at homologous lysine 120 [2]. There are two Rad6 homologs HR6A and HR6B, and two Bre1 homologs RNF20 and RNF40 [2, 27] (Table 1). HR6A and HR6B act redundantly as HR6B knockout mice have wild-type levels of H2B ub1 [2]. RNF20 and RNF40 heterodimerize to form a protein complex which is required for H2B ubiquitination [28]. WAC (*WW* domain-containing *a*daptor with *c*oiled-coil) was identified as a functional partner of RNF20/40 which regulates H2B ub1 [29]. During transcription, WAC targets RNF20/40 to associate with the Pol II complex to control H2B ubiquitination and transcription [29] (Table 2). In *Arabidopsis* (*A. thaliana*), H2B is monoubiquitinated at Lys-143 by two RING E3 ligases HUB1 and HUB2, and three E2 conjugases UBC1, UBC2, and UBC3 [30] (Table 1). Collectively, although H2B ub1 writers are highly conserved through evolution, other associated factors appear to be more specific since no sequence similarity could be found among H2B ub1-related factors Lge1, Shf1 and WAC [31]. Additionally, our study of the cereal crops pathogen *Fusarium graminearum* showed that FgBre1 contains a basic region/leucine zipper (bZIP) domain mediating target DNA-binding, which is not conserved in Bre1 orthologs from other eukaryotes [32], indicating the possibility of species-specific H2B ub1 regulation. In the last decade, H2B ub1 has been reported to be involved in several molecular and cellular functions including regulation of transcription initiation and elongation [17, 33], DNA damage response and repair [34–36], DNA replication [37], nucleosome stabilization [10–12], and heterochromatin silencing [8, 9]. Notably, H2B ub1 is responsible for inducible rather than constitutive transcription regulation, and is associated with transcription regulation of specific genes groups rather than the whole genome [4, 27, 38]. However, how H2B ub1 is induced and precisely enriched to the target chromosome and whether above H2B ub1 related factors contribute to the precise H2B ub1 enrichment to target chromosome remain unclear.

**Table 1:** Enzymes mediating H2B mono-ubiquitination and deubiquitination

Species	H2B mono-ubiquitination		H2B deubiquitination	Reference
	E2	E3	DUBs	
*S. cerevisiae*	Rad6	Bre1	Ubp8, Ubp10	[20, 21, 27, 37]
*S. pombe*	Rhp6	Brl1, Brl2 (Rfp2, Rfp1)	Ubp8	[8, 39]
Mammals	HR6A, HR6B	RNF20, RNF40	USP3, USP7, USP12, USP22 USP44, USP46, USP49	[5, 26, 27, 40–44]
*A. thaliana*	UBC1, UBC2, UBC3	HUB1, HUB2	SUP32, UBP26	[28, 45]

**Table 2:** Proteins associated with H2B mono-ubiquitination and deubiquitination

Protein	Species	Function	Reference
Lge1	*S. cerevisiae*	Cofactor of Bre1 required for the full level of H2B ub1	[18]
Bur1	*S. cerevisiae*	Kinase together with Bur2 required for H2B ubiquitination	[22]
Bur2	*S. cerevisiae*	Cyclin together with Bur1 required for H2B ubiquitination	[22]
Rtf1	*S. cerevisiae*	Component of PAF complex regulating Bre1 stability	[23]
Shf1	*S. pombe*	Serine-rich protein within HULC complex necessary for H2B ubiquitination	[8]
Mst2	*S. pombe*	A member of the MYST family acetylating Brl1	[25]
WAC	Mammals	Functional partner of RNF20/40 promoting RNF20/40 ligase activity for H2B ubiquitination	[2]
SPT20	*S. cerevisiae*	Component of SAGA complex required for the integrity and deubiquitination activity of SAGA complex	[37]
Sgf1	*S. cerevisiae*	Component of DUB module in SAGA complex required for the deubiquitination activity of SAGA complex	[40, 46]
Sus1	*S. cerevisiae*	Component of DUB module in SAGA complex required for the deubiquitination activity of SAGA complex	[40, 46]
Sgf73	*S. cerevisiae*	Component of DUB module in SAGA complex required for the deubiquitination activity of SAGA complex	[40, 46]

### H2B deubiquitination

‘Editors’ are enzymes that remove or modify histone modifications [5]. Ubiquitin can be removed from H2B by ubiquitin-specific proteases defined as deubiquitination enzymes (DUBs) [5, 6]. In *S. cerevisiae*, several H2B DUBs have been identified, the main ones being Ubp8 and Ubp10 (Dot4) [40, 46] (Table 1). Ubp8, is a component of the Spt-Ada-Gcn5-acetyltransferase (SAGA) complex and responsible for H2B deubiquitination both in vitro and in vivo [46, 47]. SAGA complex integrity is required for Ubp8 deubiquitination activity. Disrupting *SPT20*, which is required for SAGA integrity, resulted in increased levels of H2B ub1(Table 2), although the H2B ub1 level in *SPT20* deletion mutant was slightly lower than that in *UBP8* deletion mutant, indicating Ubp8 targets H2B ub1 for hydrolysis primarily as a component of SAGA [48]. Furthermore, Ubp8 bound to Sgf11, Sus1 and Sgf73 in a distinct subcomplex called the deubiquitinating module (DUBm) in the SAGA complex (Table 2), and the deubiquitinating activity depends upon the presence of all four DUBm proteins [39, 49]. Ubp10 is another H2B ub1 protease functioning independently of SAGA complex. A Ubp10 deletion mutant showed increased H2B ub1 level, similar as *UBP8* deletion mutant (Table 1). Deletion of both *UBP10* and *UBP8* results in a synergistic increase in the steady-state levels of H2B ub1 and in the number of genes with altered expression, indicating that these two ubiquitin proteases likely overlap in some of their target chromatin regions and may cooperate to regulate the global balance of H2B ub1. The deubiquitination activity of Ubp8 is highly conserved as its orthologs are identified in *S. pombe* (Ubp8), *F. graminearum* (Upb8), *Drosophila melanogaster* (Nonstop) and humans (USP22) [41, 45] (Table 1), while no Ubp10 ortholog was found in *S. pombe* or metazoans. In *F. graminearum*, Upb10 ortholog exists but does not regulate the level of H2B ub1 [50]. In *Drosophila*, phylogenetic analysis demonstrates that Ubp10 homolog might be CG15817 [44], while another deubiquitinase USP7 has been implicated in catalyzing deubiquitination of H2B ub1 in vitro [42] (Table 1). In *Arabidopsis*, loss of H2B deubiquitinase SUP32/UBP26 increases the levels of H2B ub1 [43] (Table 1). In mammals, USP3, USP7, USP12, USP22, USP44, USP46 and USP49 have all been reported to be involved in H2B deubiquitination [5, 45, 51–53] (Table 1). However, how these editors function in maintaining the dynamic equilibrium between H2B ubiquitination and deubiquitination, and how they compete with H2B ub1 writers for access to a specific region on the nucleosome or target gene chromosome to modulate H2B ub1 levels and target gene transcription remain to be studied.

## Functional mechanisms of H2B ub1/deub1

### Regulation of chromatin dynamics and nucleosome stability during transcription

**Fig. 1:**
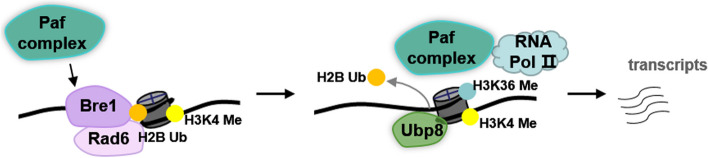
Model of dynamic H2B ub1 state in the transcription regulation

H2B ub1 has been reported to be required for expression of specific inducible genes, which is consistent with its role in transcriptional activation [7]. However, recent studies investigating the effect of H2B ub1 on chromatin structure demonstrated that, in addition to the supposed role for H2B ub1 in opening up chromatin, H2B ub1 is important for nucleosome stability [11]. Firstly, given that ubiquitin is a bulky modification and H2B ub1 is closely associated with transcriptionally active regions, H2B ub1 was originally postulated to act as a “wedge” which non-specifically opens up the chromatin and allows access to the histone modifying enzymes for subsequent transcription activation (such as its trans-histone regulation of transcription activation markers histone H3 lysine 4 and lysine 79 methylations) [20, 54]. However, (Mahesh’s group) when the “Wedge” model was tested by substituting ubiquitin with a bulkier SUMO moiety at the H2B C-terminal region in budding yeast, it was found that although the occupancy and distribution of the induced H2B sumoylation on chromatin were similar to H2B ub1, the sumoylation at the H2B C-terminus cannot functionally replace ubiquitination to support H3K4 and -K79 methylations. Thus, the SUMO or ubiquitin moiety, do not act as a mere ‘wedge’’ to open up chromatin structure [55]. Secondly, micrococcal nuclease (MNase) assays were performed to test the effect of H2B ub1 on overall chromatin structure using wild-type yeast strain and mutants ΔRad6, ΔBre1 and H2B-K123R completely lacking H2B ub1, and mutants ΔUbp8 and ΔUbp10 showing high levels of H2B ub1. The results showed that the histone occupation rate on native chromatin was increased in the presence of high levels of H2B ub1, indicating that presence of H2B ub1 makes the chromatin compact, while the absence of H2B ub1 leads to an “open” or loose chromatin [55]. This was supported by the observation that in vitro reconstituted nucleosomes containing H2B ub1 showed a significantly lower DNase I digestion sensitivity compared to H2B without ubiquitination [11]. Fourthly, structure analysis of ubiquitin showed that the charged residues (one-third of the molecule) of ubiquitin are on the surface, which modulate nucleosome stability via its direct contact with the DNA and/or other core histones and subsequently stabilizing the intra- and inter-chromatin array contacts [55]. In summary, H2B ub1 state is closely associated with chromatin dynamics, and contrary to the supposed role for H2B ub1 in opening up chromatin, it is important for nucleosome stability. These observations raise the question, how H2B ub1 functions in stabilizing nucleosomes, restricting nuclease accessibility while facilitating RNA polymerase II (Pol II) elongation and transcription activation. One hypothesis proposed here is that H2B ub1 initially stabilizes the nucleosomes to enhance elongation factor or histone methyltransferase to chromatin next to the polymerase, then Ubp8 mediating deubiquitination to destabilize the nucleosomes and allow Pol II progression (Fig.1). Consisting with our hypothesis, it was found that the absence of H2B ub1 resulted in a reduction of chromatin-bound elongation factor Spt16 [56]. Moreover, H2B ub1 is reported to be required for the association of H3K4 methyltransferase complex COMPASS key component for subsequent H3K4 methylation and transcription process [57], indicating the possibility of H2B ub1 may stabilizes the nucleosomes to enhance other factors binding for transcription activation. Additionally, Karl’s group found that level of H2B ub1 at the TATA region of yeast *GAL1* gene increased and then decreased during gene activation and they demonstrated a model illustrating the effects of H2B ub1 states in gene induction. In normal gene induction which is accompanied by sequential H3 K4 and K36 methylation, H2B ub1 is transient, increasing and then decreasing to promote activation. H2B ub1 helps to establish H3K4 methylation at the TATA region, then Ubp8 mediating the H2B deubiquitination maintains the normal balance between K4 and K36 methylation of H3, subsequently inducing the transcription activation [48]. Taken together, H2B ub1 level is dynamic during transcription, it may initially increase the stability of nucleosomes to enhance elongation factor or histone methyltransferase to chromatin next to the polymerase, then Ubp8 mediating deubiquitination to maintain the H3 methylation balance as well as destabilize the nucleosomes, allowing Pol II progression finally (Fig.1). However, there is still lack of direct evidence to explain how H2B ub1 function in stabilizing nucleosomes while mediating transcription activation.

### The trans-histone crosstalk between H2B ub1 and H3 methylations during transcription activation

Among all H2B ub1-regulated processes, one of best studied is its involvement in a trans-tail regulation of H3K4 and H3K79 methylation [5, 31, 33]. H3K4 and H3K79 methylations are two marks associated with active transcription. The transcriptional impact of H2B ub1 partly stems from its regulation of H3 methylation (me2/3) at K4 and K79, essential for many cellular processes including growth, development, pathogenesis and aging [12, 32, 54, 56, 58]. Additionally, these trans-tail regulations are highly conserved from yeast, fungi, to higher eukaryotes, and function indirectly as neither deletion of the relevant methyltransferases, nor mutation of the H3 methylation sites has reciprocal effect on H2B ubiquitination [32, 33].

Whereas metazoans containing multiple H3K4 methyltransferase complexes (COMPASS/MLL/COMPASS-like complexes), budding yeast only contains the COMPASS complex, which provides a good model system for studying H3K4 methylation. The COMPASS complex comprises eight subunits: Set1, Bre2 (Cps60), Swd1 (Cps50), Spp1 (Cps40), Swd2 (Cps35), Swd3 (Cps30), Sdc1 (Cps25), and Cps15 [59, 60], and all components are involved in H3K4 methylation except Cps15 [15] Several models have been proposed for the molecular mechanisms associated with H2B ub1 and H3K4 methylation. In 2007, Jung-Shin et al. showed that H2B ub1 facilitates the association of Swd2 (Cps35) with chromatin in yeast, thus regulating the occurrence of H3K4 methylation [57]. Later in 2008, Vitaliano-Prunier et al. confirmed the crucial role of Swd2 in mediating this crosstalk, however, in striking contrast, they found H2B ub1 did not affect the incorporation of Swd2 into COMPASS complex. Instead, the ubiquitination at Lys 68 and Lys 69 of Swd2 catalyzed via H2B ub1 ubiquitination enzymes Rad6/Bre1 is responsible for the recruitment of Spp1, thus subsequently affecting H3K4 me3 [13]. However, in recent studies, results demonstrate that the catalytic region of COMPASS encompassing Set1, Swd1, Swd3, Spp1, Bre2 and Sdc1, but lacking Swd2 and Shg1, possesses H2B ub1-dependent H3K4 methylation activity in yeast [15, 61], and H2B ub1-induced conformational changes in the catalytic region result in altered catalytic properties of COMPASS [15]. Consistently, cryoelectronic microscopy analysis demonstrated that an arginine-rich motif (ARM) in the nSET domain of SET1 inhibits COMPASS catalytic activity, and H2B ub1 overrides the inhibitory effect of the Set1 ARM helix by allosterically modulating the packing of the Set1 catalytic domain against Swd1, Bre2, and Swd3 [16]. However, contrarily, research in human MLL complex reveled that without the ARM of the nSET domain, the COMPASS catalytic region also has detectable response to H2B ub1 [62]. Thus, despite extensive efforts, related mechanisms for this trans-tail regulation remain elusive.

As for the crosstalk between H2B ub1 and H3K79 methylation, a model called the “Bcrash barrier” has been developed that describes the mechanism of this crosstalk based on in vitro data in yeast and mammalian cells. In this model, the ubiquitin folds back onto the nucleosome and forms a barrier to H3K79 methyltransferase Dot1, increasing the chance that it is well-positioned for methylating H3K79 [63]. However, further in vivo studies are needed to obtain more direct evidence supporting this model. Additionally, another study in yeast found that H2B ub1 is required for the association of Swd2 with the COMPASS complex, and Swd2 was proposed to interact with Dot1, then affect H3K79 methylation, which might also link H2B ub1 to H3K79 methylation [57]. To sum up, these H3 methylations governed by H2B ub1 play essential roles in cellular processes, but the corresponding regulation mechanisms are unclear and we by now are only at the beginning of understanding these trans-tail regulations.

### Roles of H2B ub1/deub1 in transcription elongation

Transcription regulated by Pol II is commonly divided into three major phases: initiation, elongation, and termination [64]. Transcription elongation of eukaryotic genes includes both the early steps of Pol II pausing and the release of Pol II entering productive elongation [59, 60]. Histone modifications and histone chaperones can loosen or tighten the interaction between DNA and nucleosomes directly, leading to the promotion or restriction of Pol II transcription [56, 65, 66]. Accessory elongation factors can facilitate Pol II movement through chromatin by controlling specific processes such as alleviating pausing and stalling during elongation [59, 67]. Furthermore, thermodynamically stable sequences in the genome may be more difficult to be transcribed, thereby affecting elongation rates [68]. H2B ub1 has linked to transcriptional elongation in *S. cerevisiae* [17, 56]. It enhances the rate of elongating Pol II recruitment to the coding sequence of an inducible yeast gene, *GAL1*, indicating that H2B ub1 might be directly required for transcription by Pol II. H3K4 methylation does not alter the rate of elongating Pol II recruitment at* GAL1*, demonstrating the function of H2B ub1 in regulation of transcriptional elongation is independent of H3K4 methylation in vivo [69]. In *S. pombe*, the observation that Pol II occupancy is reduced at the 3’ end of coding sequences in the H2B-K119R mutant also supports this finding [70]. In mammals and yeasts, several studies have identified genetic and functional interactions between the H2B ub1/deub1 enzymes and transcription elongation regulators [33, 40, 47], mainly suggesting its role in chromatin structure regulation and stage-specific process control during transcription elongation.

Polymerase associated factor 1 complex (Paf1C), which consists of Paf1, Ctr9, Leo1, Rtf1, and Cdc73, is associated with initiating and elongating of Pol II in humans and *S. cerevisiae* [71, 72]. It facilitates the deposition of H2B ub1 by directly interacting with Rad6 via its Rtf1 subunit [73, 74]. Deletion or knockdown of Paf1C reduces global levels of H2B ub1 [75]. In the Rtf1 mutant, Rad6 is still recruited to the promoter during transcription initiation, but it fails to move into the open reading frame, indicating that the Paf1C is required for Rad6 to travel with elongating Pol II [76]. The positive transcription elongation factor-b (P-TEFb) mediates the release of paused Pol II [77, 78]. In baker yeast, two P-TEFb homologs exist and both are linked to H2B ub1. One of them is Bur1 kinase/Bur2 cyclin, which is similar to Paf1C, being identified as a regulator of H3K4 methylation and shown to be required for H2B ub1 [23, 79]. Deletion of Bur2 impaired the recruitment of Paf1C, suggesting that Bur1/Bur2 together with Paf1C is responsible for recruiting Rad6/Bre1. Another P-TEFb homolog is Ctk1/Ctdk-I, and its recruitment was negatively regulated by H2B ub1, in which H2B ub1 was proposed to act as a barrier [80]. In this model, removal of H2B ub1 by SAGA following transcription initiation would be required for Pol II CTD phosphorylation of Ser-2 and further recruitment of Set2 for consequent H3K36 methylation, which reveals the importance of H2B deub1 in transcription elongation. In summary, Bur1/Bur2 cooperates with Paf1C to facilitate H2B ubiquitination whereas Ctk1 functions sequentially during productive transcription elongation.

**Fig. 2:**
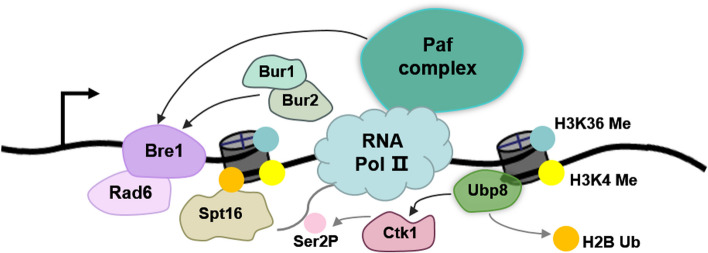
Model of transcription elongation regulation by H2B ub1/deub1 and transcription elongation factors

A hallmark of transcription elongation is the reduction in nucleosome stability that occurs during the passage of Pol II. Nucleosome dynamics can be regulated by histone-binding chaperones, which tether specific histones during nucleosome eviction or promote histone deposition [81–83]. In *S. cerevisiae*, H2B ub1 interacts with Spt16, a subunit of the histone chaperone FACT which facilitates chromatin transcription to reassemble nucleosomes after the passage of Pol II at the replication fork. This interaction is mutually reinforced as Spt16 promotes both global and local H2B ub1, and in turn, H2B ub1 promotes accumulation of Spt16 on the target chromatin region [54, 55]. Importantly, H2B ub1 also stimulates FACT-mediated displacement of H2A/H2B dimer from the core nucleosome and enhances the frequency of Pol II passage through the chromatin templates [16], which is considered to be a direct role for H2B ub1 in transcription elongation. In *Arabidopsis*, FACT genetically interacts with HUB1 to regulate multiple development processes [84]. What’s more, Paf1C interacts with FACT functionally and physically [85], thus working together to attract transcriptionally active genes. In conclusion, besides directly required by Pol II, H2B ub1-related ubiquitinating enzymes, Paf1C, histone chaperones and elongation factors were shown to cooperate with each other and promote appropriate transcription elongation (Fig. 2).

### H2B ub1 and heterochromatin silencing

Euchromatin has an open structure and contains active genes that are permissive for transcription, while heterochromatin is maintained in a compact structure to prevent transcription and silence genes. Intriguingly, heterochromatic loci have low H2B ub1 levels, indicating besides activating euchromatin transcription, H2B ub1 also plays crucial roles in nucleosome stabilization and heterochromatin modulations.

In budding yeast, heterochromatin is mediated by the silent information regulator (SIR) complex composed of Sir2, Sir3, and Sir4 at the silent mating type loci and the telomeres [86, 87]. SIR complex can deacetylate H4K16, but it cannot silence chromatin with H3 methylation present. Considering that H2B ub1 is a prerequisite for H3K4 and H3K79 methylation and positively regulates H4K16 acetylation levels near the telomeres, it indirectly antagonizes SIR complex-dependent silencing [46, 88–90]. The removal of this modification by deubiquitinating enzymes is essential for preserving of silenced chromatin structures. Ubp10 but not Ubp8 plays an important role in this process by maintaining low levels of H2B ub1 [88, 89, 91]. What’s more, Ubp10 is preferentially recruited to chromatin during assembly of the SIR complex onto chromatin rather than to an established heterochromatin, thus contributing to SIR complex-mediated heterochromatin assembly and formation [92]. However, the deubiquitination activity of Ubp10 is impeded when its substrate, ubiquitinated H2B, is assembled into a nucleosome, and whether H2B ub1 directly affects SIR complex activities on chromatin remains to be explored [9].

In fission yeast, the ubiquitin-conjugating enzyme Rhp6 was reported to be a negative regulator of heterochromatic transcriptional gene silencing. Overexpression of *RHP6* leads to disrupted silencing on account of increases in Pol II occupancy and decreases in H3K9 me3 at centromeres, whereas deletion of *RHP6* enhances heterochromatin silencing [8, 19]. Reduction of H2B ub1 level together with increases in the initiation and maintenance of silencing can be observed in the *Mst2* mutant, since histone acetyltransferase Mst2 positively regulates the activity of ubiquitin ligase Brl1 [26]. The Paf1C functions upstream in the H2B ub1 pathway, and deletion of Paf1C subunits also enhances silencing [93]. Together, reductive H2B ub1 levels impair transcription by slowing nascent transcripts release and allow the silencing machinery to target these transcripts, further promoting the integral RNAi-dependent silencing system to generate siRNA and H3K9 me3 biogenesis for heterochromatin initiation [9, 94]. But the underlying mechanisms that H2B ub1 influences heterochromatin dynamics and release from the regions undergoing silence remain unexplored.

## Roles of H2B ub1/deub1 in plant-pathogen interactions

As important epigenetic modifications, H2B ub1/deub1 have been also reported to play crucial roles in diverse life processes in plants and fungi [95, 96]. Here, we focus on the functions of H2B ub1/deub1 in the interactions of plant and pathogenic fungi. In *Arabidopsis*, mutants deficient in the ubiquitinating ligases Hub1 exhibited higher sensitivity to the necrotrophic fungal *Botrytis cinerea* and *Alternaria brassicicola*, and expression of *HUB1* is induced at the site of the infection. HUB1/HUB2 mediate H2B ub1 deposition directly at the resistance gene suppressor of npr1-1, constitutive 1 (*SNC1*)*,* subsequently induce its expression, which is associated with autoimmune phenotypes and enhanced disease resistance [97]. In response to *Verticillium dahliae* (*Vd*) toxins, H2B ub1 modulates the expression of protein tyrosine phosphatase 1 (*AtPTP1*) and atypical dual-specificity phosphatase 5 (*AtPFA-DSP5*), which results in rapid depolymerization of microtubules (MTs) through an AtPTP1-mediated signaling pathway to defend against *Vd* toxins [29]. H2B ub1 also accelerates cell death and H_2_O_2_ production in response to *Vd* toxin by increasing deposition of H3K4 me3 on the NADPH oxidase gene respiratory burst oxidase protein homologue D (*RBOHD*) [97]. The HUB1/HUB2 homologues in tomato, SlHUB1/SlHUB2, have similar H2B monoubiquitination E3 ligase activity in vitro, and positively regulate the defense response against *B. cinerea* by adjusting the balance between the salicylic acid-, jasmonic acid-, and ethylene-mediated signaling pathways [98]. Similarly, OsHUB1 and OsHUB2 of rice could form a complex with spl11-interacting protein 6 (*SPIN6*), a Rho GTPase-activating protein that negatively regulates plant cell death and innate immunity, to promote OsRac1-associated defense. They are down-regulated in the SPIN6 RNAi plants and during the compatible interaction between rice and *Magnaporthe oryzae* [99].

As a response to pathogen attack, plants usually induce oxidative, iron excess/storage and other stresses towards pathogens, and secrete antimicrobial substances such as polyamines and phytoalexins against pathogens invasion [100–104]. In *F. graminearum*, H2B ub1 was found to regulate the biosynthesis of virulence factor deoxynivalenol that is induced by host-produced putrescine during infection [32]. Moreover, both H2B ub1 and H2B deub1 participate in the adaption of host-derived iron excess during infection [50]. Altogether, the function of H2B ub1/deub1 in plant-pathogen interactions is still poorly studied, it will be interesting to determine whether the spatiotemporal and dynamic nature of pathogenic invasion is affected by H2B ub1/deub1 levels in both the pathogen and its host plants.

## Conclusions

Studies in the past two decades have greatly widened our understanding of how H2B mono-ubiquitination and deubiquitination contribute to maintaining a dynamic balance in regulating gene expression. The dynamic state of H2B ub1 determines nucleosome status, ubiquitinated H2B enhances the nucleosome compaction for other factors binding and activates transcription in the initiation phase. Then Ubp8 mediated deubiquitination destabilizes the nucleosomes and favor transcription elongation in the wake of Pol II [40]. The crosstalk between H2B ub1 and H3 methylations has been well studied and involved in many cellular processes. But in the ciliate *Tetrahymena thermophila*, lack of H2B ubiquitination did not cause detectable change in H3K4 methylations [105], indicating that the mechanisms of trans-histone crosstalk are not universal. Chromatin immunoprecipitation (ChIP)-seq analysis in *S. cerevisiae* suggested that H2B ub1 is not only distributed at the promoter but also in the transcribed region of active genes, corresponding to its role in transcription elongation [7, 76]. However, it remains to be resolved if H2B ub1 positioned along with elongating Pol II play a role in addition to the transcription process itself, such as being a signal in response to environmentally specific factors for gene expression or maintaining protein stability.

Besides mediating changes in transcription, several studies have suggested that H2B ub1 could also regulate post-transcriptional events such as mRNA processing 3’ end processing [106], and mRNA export and splicing [107] in *S. cerevisiae* and *Arabidopsis*. In animals, microarray analysis suggested a potential role for H2B ub1 also in protein translation and the regulation of protein phosphorylation [108]. Thus, we expect and believe that the recent developments in genome techniques and chromosome conformation methods will accelerate the exploration of as yet unknown roles of H2B ub1/deub1 in different cellular processes, and to unravel genome-wide epigenetic changes and complex regulatory networks underlying this dynamic process.

## Data Availability

All data used in this study are included in this article.
